# Assessing CT-based volumetric analysis via deep learning for idiopathic normal pressure hydrocephalus

**DOI:** 10.1093/braincomms/fcag300

**Published:** 2026-08-05

**Authors:** Meera Srikrishna, Woosung Seo, Anna Zettergren, Silke Kern, Daniel Cantré, Florian Gessler, Houman Sotoudeh, Jakob Seidlitz, Joshua D Bernstock, Lars-Olof Wahlund, Eric Westman, Ingmar Skoog, Johan Virhammar, David Fällmar, Michael Schöll

**Affiliations:** Wallenberg Centre for Molecular and Translational Medicine, University of Gothenburg, Gothenburg 40530, Sweden; Department of Psychiatry and Neurochemistry, Institute of Physiology and Neuroscience, University of Gothenburg, Gothenburg 40530, Sweden; Department of Surgical Sciences, Neuroradiology, Uppsala University, Uppsala 75185, Sweden; Neuropsychiatric Epidemiology, Institute of Neuroscience and Physiology, Sahlgrenska Academy, Centre for Ageing and Health (AgeCap), University of Gothenburg, Gothenburg 43141, Sweden; Neuropsychiatric Epidemiology, Institute of Neuroscience and Physiology, Sahlgrenska Academy, Centre for Ageing and Health (AgeCap), University of Gothenburg, Gothenburg 43141, Sweden; Department of Psychiatry and Neurochemistry, Institute of Neuroscience and Physiology, Sahlgrenska Academy, University of Gothenburg, Mölndal 43141, Sweden; Region Västra Götaland, Department of Neuropsychiatry, Sahlgrenska University Hospital, Gothenburg 40530, Sweden; Institute of Diagnostic and Interventional Radiology, Pediatric Radiology and Neuroradiology, University Medical Center Rostock, Rostock 18057, Germany; Department of Neurosurgery, University Medicine of Rostock, Rostock 18057, Germany; Department of Radiology, UT Southwestern, Dallas, TX 5323, USA; Lifespan Brain Institute, The Children’s Hospital of Philadelphia and Penn Medicine, Philadelphia, PA 19104, USA; Institute for Translational Medicine and Therapeutics, University of Pennsylvania, Philadelphia, PA 19104-5158, USA; Department of Psychiatry, University of Pennsylvania, Philadelphia 19104, USA; Department of Child and Adolescent Psychiatry and Behavioral Science, The Children's Hospital of Philadelphia, Philadelphia 19104, USA; Department of Neurosurgery, Brigham and Women’s Hospital, Harvard Medical School, Boston, MA 02115, USA; David H. Koch Institute for Integrative Cancer Research, Massachusetts Institute of Technology, Cambridge, MA 02139, USA; Division of Clinical Geriatrics, Department of Neurobiology, Care Sciences and Society, Karolinska Institutet, Stockholm 17177, Sweden; Division of Clinical Geriatrics, Department of Neurobiology, Care Sciences and Society, Karolinska Institutet, Stockholm 17177, Sweden; Neuropsychiatric Epidemiology, Institute of Neuroscience and Physiology, Sahlgrenska Academy, Centre for Ageing and Health (AgeCap), University of Gothenburg, Gothenburg 43141, Sweden; Department of Medical Sciences, Neurology, Uppsala University, Uppsala 751 85, Sweden; Department of Surgical Sciences, Neuroradiology, Uppsala University, Uppsala 75185, Sweden; Wallenberg Centre for Molecular and Translational Medicine, University of Gothenburg, Gothenburg 40530, Sweden; Department of Psychiatry and Neurochemistry, Institute of Physiology and Neuroscience, University of Gothenburg, Gothenburg 40530, Sweden; Region Västra Götaland, Department of Neuropsychiatry, Sahlgrenska University Hospital, Gothenburg 40530, Sweden

**Keywords:** CT, MRI, hydrocephalus, segmentation, deep learning

## Abstract

Brain computed tomography (CT) is an accessible and commonly utilized technique for assessing brain structure. In cases of idiopathic normal pressure hydrocephalus (iNPH), the presence of ventriculomegaly is often neuroradiologically evaluated by visual rating and manual measurement of each image. Previously, we have developed a deep-learning-model that utilizes transfer learning from magnetic resonance imaging (MRI) for CT-based intracranial tissue segmentation. Accordingly, herein we aimed to enhance the segmentation of ventricular cerebrospinal fluid (VCSF) in brain CT scans and assess the performance of automated brain CT volumetrics in iNPH patient diagnostics.

This retrospective study employed a two-stage approach in developing the model. Initially, a 2D U-Net model was trained to predict VCSF segmentations from CT scans, using paired MR-VCSF labels from healthy controls. This model was subsequently refined by incorporating manually segmented lateral CT-VCSF labels from iNPH patients, building on the features learned from the initial U-Net model. The training dataset included 734 CT datasets from healthy controls paired with T1-weighted MRI scans from the Gothenburg H70 Birth Cohort Studies and 62 CT scans from iNPH patients at Uppsala University Hospital. To validate the model's performance across diverse patient populations, external clinical images including scans of 11 iNPH patients from the Universitätsmedizin Rostock, Germany, and 30 iNPH patients from the University of Alabama at Birmingham, United States were used. Further, we obtained three CT-based volumetric measures (CTVMs) related to iNPH.

Our analyses demonstrated strong volumetric correlations (*ρ* = 0.91, *P* < 0.001) between automatically and manually derived CT-VCSF measurements in iNPH patients. Based on the ventricular volume, the CTVMs exhibited high accuracy in differentiating iNPH patients from controls in external clinical datasets with an AUC of 0.97 (95% CI: 0.94–1.00) and in the Uppsala University Hospital datasets with an AUC of 0.99 (95% CI: 0.98–1.00).

CTVMs derived through deep learning show potential for assessing and quantifying morphological features in hydrocephalus. Critically, these measures performed comparably to gold-standard neuroradiology assessments in iNPH patients and healthy controls, even in the presence of intraventricular shunt catheters. Accordingly, such an approach may serve to improve the radiological evaluations of the diagnostic work-up and treatment response monitoring in patients with hydrocephalus. Since CT is much more widely available than MRI, our results have considerable clinical impact.

## Teaser

Srikrishna *et al*. report that automated CT analysis accurately identifies imaging features of idiopathic normal pressure hydrocephalus. A deep learning model trained using MRI and manually derived labels performed strongly across multiple cohorts, supporting its potential to aid diagnosis and reduce subjectivity in radiologic assessment.

## Graphical Abstract

**Graphical Abstract:**
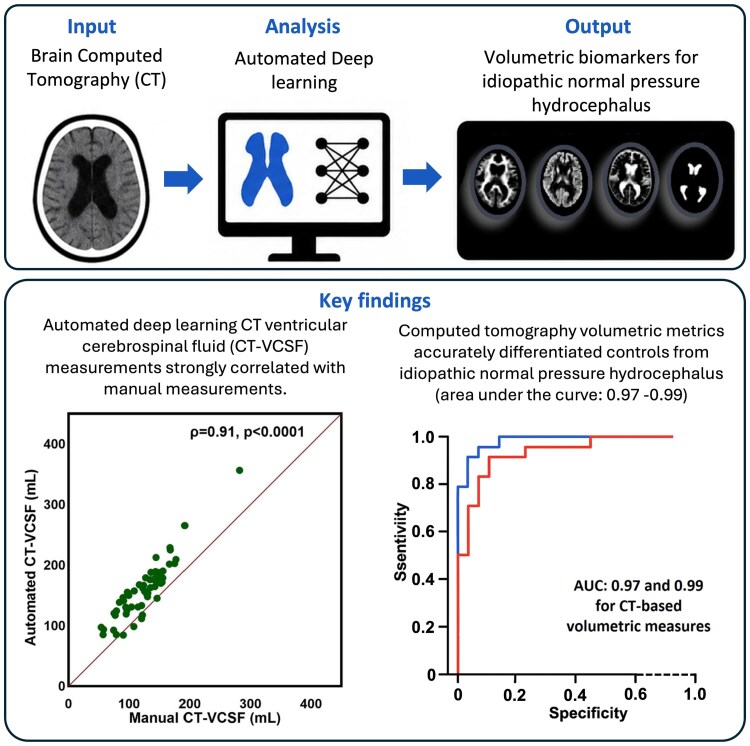

## Introduction

Idiopathic normal pressure hydrocephalus (iNPH) is a treatable neurological condition associated with a triad of clinical symptoms consisting of gait and balance issues, cognitive impairment, and urinary incontinence.^1,2^ A recent scientific discourse has suggested the term ‘Hakim disease,’ but a consensus on its use has not yet been reached.^3^ The standard treatment for iNPH involves neurosurgical diversion of cerebrospinal fluid (CSF) via the placement of an intraventricular shunt.^2,4^ The estimated prevalence of iNPH is approximately 1.5–3.7% among individuals aged 65 and older, rising to 4–6% in those aged 80 and above.^5,6^ The condition is strongly associated with enlarged ventricles in combination with certain morphological features, and the work-up of suspected iNPH includes structural brain imaging with CT or MRI.^2^ Those who meet clinical and radiological criteria are usually referred for further assessments at a specialized centre, where candidates for shunt surgery are selected.^7,8^

Since the clinical presentation is often difficult to separate from age-related processes and other diseases in an elderly population, radiological features of iNPH are essential for pre-selecting adequate patients for further work-up. In addition to visual assessment of ventriculomegaly, typical radiological markers include the Evans’ index^9^ (EI), callosal angle,^10,11^ and disproportionately enlarged subarachnoid spaces.^12^ These measurements rely on assessments made in the bi-commisural plane.^13^ The EI is defined as the ratio between the maximal width of the frontal horns of the lateral ventricles and the maximum inner diameter of the skull. While EI provides a coarse and simplified approximation of ventricular size, it only measures a single diameter in a particularly three-dimensional structure. It also lacks specificity among the elderly population as it does not provide information about cerebral atrophy.^14,15^

Truly volumetric measurements offer a much more precise method for assessing ventricular size, and has recently become more easily available due to advances in image processing. In line with this, research has shown that three-dimensional analysis of ventricular volume, achieved through semi-automated volumetric techniques or atlas-based segmentation, is more sensitive than the Evans’ index for monitoring ventricular size changes after shunt treatment in patients with iNPH.^16-18^ Volumes of ventricular cerebrospinal fluid (VCSF) are currently measured using automated or manual segmentation, but only in research environments. MRI, known for its excellent soft-tissue contrast, offers superior potential for automated segmentations and identification of anatomical landmark.^19^ From an image quality perspective, it is therefore the preferred method for automated quantification of ventricles and other brain tissue types.^20-23^

Conversely, computed tomography (CT) offers a more accessible and cost-effective alternative, with the advantages of shorter scan durations, broader availability, and the absence of strong magnetic fields. Moreover, CT scans are routinely used in the clinical work-up of elderly with falling accidents (head trauma), and suspected neurodegenerative disorders (e.g. cognitive issues). The frequent clinical use of CT offers the opportunity to detect incidental morphological changes related to iNPH, potentially enabling earlier diagnosis and subsequent treatment of the condition.^24^ Despite these advantages, current radiological assessments still largely depend on subjective visual rating methods that are time-consuming, require significant expertise, and are highly susceptible to variability in inter-rater reliability.^11^ When evaluating post-treatment follow-up, MRI scans are also affected by artefacts around the site of the implanted shunt valve, which limits automatic volumetry. Accurate volumetric assessment in the presence of a shunt is potentially useful for radiological monitoring of treatment response but requires robust segmentation methods.

While numerous studies have successfully demonstrated brain tissue segmentation and volumetric assessment using MR images,^25,26^ these techniques have encountered challenges when applied on CT scans, due to inferior soft tissue contrast. Recent research has explored robust automated and semi-automated segmentations^27-29^ and volumetric assessments of CT images using state-of-the-art image processing techniques centred on deep learning. Specifically, segmentation models that employ deep learning architectures, such as fully connected convolutional neural networks and 2D/3D U-Nets, have been utilized.^30-33^ So far, most deep-learning-based studies have been conducted on limited datasets and relied on manually annotated structural labels.

In our previous research, we successfully employed MR-based tissue class segmentations, derived automatically using established and validated methods, to train deep learning models for tissue classification in brain CT scans.^34^ To accomplish this, we trained U-Net-based deep learning models to segment grey matter, white matter, CSF, and intracranial volume (ICV) in brain CT images with slice thicknesses ranging from 3 to 5 mm.^34^ Leveraging our expertise and recognizing the underused potential of CT in diagnosing and managing neurological disorders such as iNPH globally, our current goal is to develop deep learning models trained on both manually and automatically derived labels. These models were designed to segment VCSF, specifically targeting the lateral ventricles in brain CT images, using transfer learning techniques that are optimal for scenarios with limited training data.^35,36^

Furthermore, we aimed to extract CT-based volumetric metrics (CTVMs) related to iNPH using deep learning. We evaluated their performance in differentiating hydrocephalus (i.e. iNPH patients) from controls. Finally, we quantified ventricular cerebrospinal fluid (VCSF) volume before and after shunt surgery in iNPH patients to enable automated monitoring of treatment efficacy.

We ultimately derived three volumetric ratios. The first was the ratio of lateral ventricular volume to intracranial volume (VCSF/ICV), also known as the 3D Evans’ index (3D-EI). This metric represents a three-dimensional extension of the traditional Evans’ index and may offer improved sensitivity and precision. The second ratio was brain volume to intracranial volume (BV/ICV), sometimes referred to as the brain parenchymal fraction, which reflects the degree of overall parenchymal atrophy. The third ratio was ventricular volume to total CSF volume (VCSF/CSF), providing a more direct measure of hydrocephalus. We propose that these volumetric parameters can be used to enhance the clinical usefulness of CT images, especially for rapid and objective detection of hydrocephalus and ultimately other neurodegenerative disorders.

## Materials and methods

**Figure 1:**
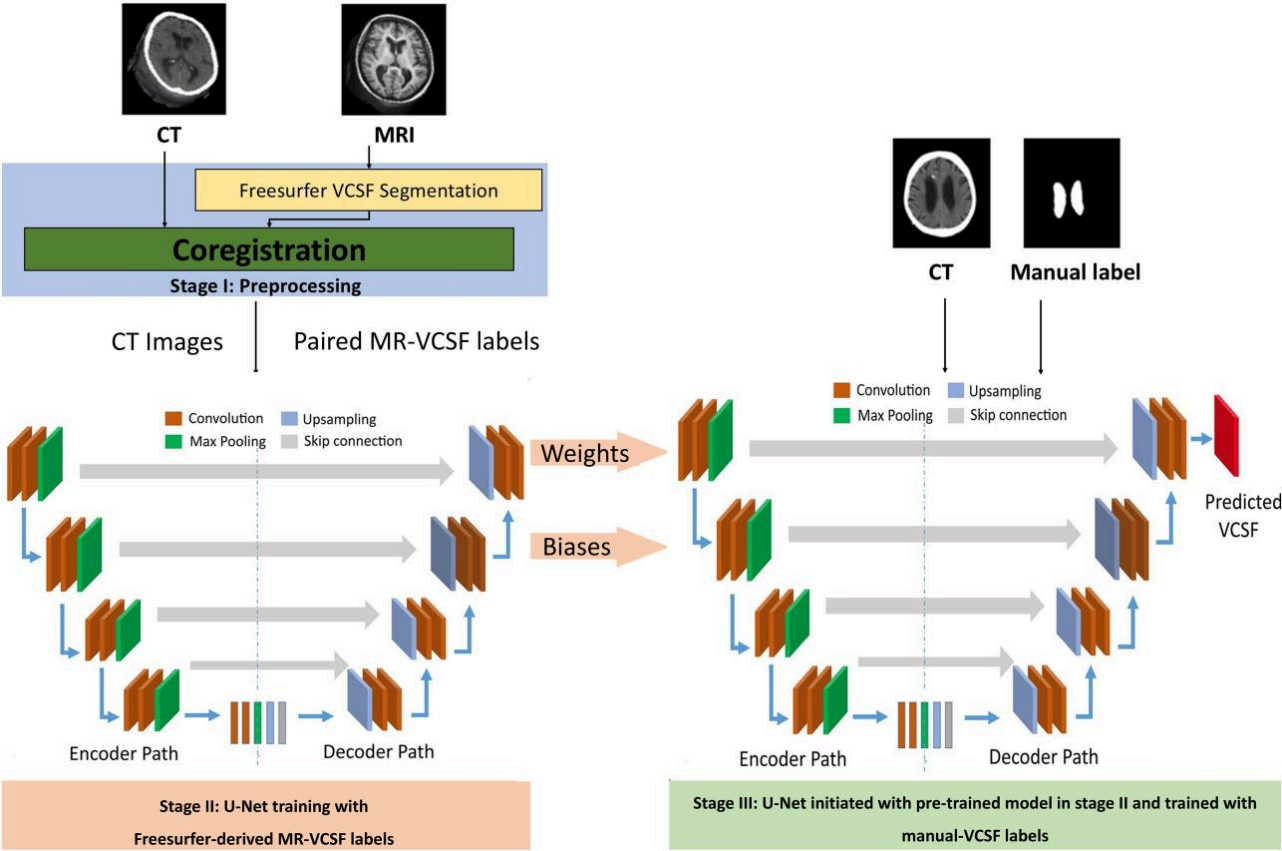
**Overview of model development and training.** Stage I: In the pre-processing stage, 734 CT-MRI pairs of 70-year-old individuals from the Gothenburg H70 Birth Cohort Studies were coregistered. MR images were segmented to lateral ventricular cerebrospinal fluid (VCSF) using FreeSurfer. Stage II: Paired CT- and MR-VCSF labels were split into training, validation, and unseen test datasets for a three-fold cross-validation. A 2D U-Net-based deep-learning model was developed and trained to predict lateral VCSF with paired CT images and MR labels as training inputs. Stage III: The weights and biases from Stage II deep-learning models were used to initialize another U-Net-model, which was further trained with paired CT and manual CT-VCSF labels from 62 iNPH patient datasets. Abbreviations: CT, computed tomography; MR, magnetic resonance; lateral ventricular cerebrospinal fluid, VCSF.

We developed models based on deep learning capable of segmenting lateral VCSF from brain CT images. These models were trained using a transfer learning approach, incorporating both automatically derived MR labels and manually derived CT labels. The model was developed in three stages: pre-processing, training the deep-learning-model with MR-based labels, and training the deep-learning model with manually derived CT labels. In this pipeline, we initialized the latter model using transferable features from the MR-VCSF label-trained pre-trained model. Figure 1 provides an overview of the model development pipeline.

### Datasets

*Gothenburg H70 Birth Cohort Studies:* The datasets used for training and validating the models obtained through deep learning (via MR-derived labels) were obtained from the predominantly healthy participants within the Gothenburg H70 Birth Cohort Studies.^37^ These multidisciplinary longitudinal epidemiological studies involve six birth cohorts, each initially enrolled and examined at the age of 70, to investigate the ageing population in Gothenburg, Sweden. Data were collected from 2014 to 2016. The H70 study was approved by the Regional Ethical Review Board and by the Radiation Protection Committee in Gothenburg, Sweden. Brain imaging was conducted at Aleris Röntgen Annedal in Gothenburg (Aleris Healthcare AB, Stockholm, Sweden). CT images were available for *n* = 917 participants, of which the vast majority (99%; *n* = 904) were cognitively normal. Of these 917 participants, 79% (*n* = 744) underwent MR scanning within a day of their respective CT scan; for model development, we included these ∼same-day acquisitions of CT and MR images from the respective 744 participants (52.6*%* female, 70.44 ± 2.6 *years*, cognitively normal, *n* = 722). CT scanning was conducted using a 64-slice Philips Ingenuity CT system (Philips Medical Systems, Best, Netherlands); resultant images had a slice thickness of 0.9 mm, an acquisition matrix of 512 × 512 and voxel size 0.5 × 0.5 × 5.0 mm^3^. MR scanning was conducted on a 3-Tesla Philips Achieva system (Philips Medical Systems). The protocol included the acquisition of MPRAGE T1-weighted images with a field of view of 256 × 256 × 160, voxel size: 1 × 1 × 1 mm^3^, echo time: 3.2 ms, repetition time: 7.2 ms, flip angle: 9*°*.

*Uppsala University Hospital:* For the deep learning models trained with manually annotated labels, this dataset included 62 CT scans from 33 iNPH patients (79.3 ± 6.46 years, 51*%* female) at Uppsala University Hospital. All patients underwent a comprehensive neurological evaluation, underwent surgical treatment with shunt insertion, and demonstrated postoperative clinical improvement as assessed by the Swedish iNPH scale. The project was approved by the regional ethics committee (Regionala etikprövningsnämnden of Uppsala, Dnr 2015/174/1-4, amended in Dnr 2022-01862-02). Manual segmentations/labels of the lateral ventricles were curated for the CT scans. Out of all patients, 23 had post-shunt scans available that featured intraventricular catheters. Three raters performed manual segmentation under the guidance of a board-certified neuroradiology specialist (DF). Manual segmentations were generated using a semiautomatic area-filling software tool (Slicevault, version Aug 2022) that allowed adjustment of pixel intensity thresholds. These were performed on the same axial image reconstructions as the automatic segmentations, under the supervision of a board-certified neuroradiologist (DF) to ensure anatomical accuracy. Visual quality control was conducted on 3D renderings of the segmented structures using ITK-SNAP (version 4.2.2).

*Clinical Validation Datasets:* To validate the diagnostic performance of the CT-based volumetric measures developed using deep learning, an external dataset was assembled. It included 11 iNPH CT scans from Universitätsmedizin Rostock (74.5 ± 7.39 *years*, 21*%* female) and 30 iNPH scans from the University of Alabama at Birmingham (70.4 ± 9.1 *years*, 33.33*%* female). All cases from the University of Alabama at Birmingham were clinically diagnosed with probable iNPH and subsequently underwent shunt surgery. For the cases from Universitätsmedizin Rostock, diagnoses were made according to the guidelines of the German Society of Neurology.^38^

### Model development

#### Pre-processing

Images were pre-processed using SPM12 (http://www.fil.ion.ucl.ac.uk/spm, MATLAB 2023a). All CT and MR images, and CT-derived manual segmentations were converted to NIfTI format(s). In the Gothenburg H70 Birth Cohort, the images were visually assessed, and the AC-PC planes were aligned. VCSF segmentations were derived from MR images using Freesurfer 5.3.0^39^ processed through the HiveDB system.^40^ Freesurfer segmentations were visually inspected on the MRI images to ensure accuracy and correct labelling. To train the model in the CT space, the MR labels were co-registered to CT images in SPM12^41^ using rigid body transformation. The registration between output CT and MR labels of each dataset was visually assessed; 10 datasets were found to have faulty co-registrations and were discarded.

#### Model development using automatically derived MR labels

At this stage, the deep learning models were trained using MR-VCSF labels. The 734 pre-processed datasets from the Gothenburg H70 Birth Cohort, consisting of CT images and FreeSurfer-derived MR-VCSF labels, were partitioned into three disjoint subsets (A, B, and C). We employed a three-fold cross-validation scheme in which three separate models were trained rotationally. Model 1 was trained on subsets A and B and evaluated on subset C; Model 2 was trained on subsets B and C and evaluated on subset A; and Model 3 was trained on subsets C and A and evaluated on subset B. In this configuration, each dataset served as an unseen test set exactly once, ensuring that no model was evaluated on data used during its training. This procedure is equivalent to a three-fold cross-validation and provides a robust estimate of model performance while mitigating potential training bias. Moreover, given the limited dataset size, this approach maximizes the use of available data for training and validation without compromising the strict separation between training and test sets. For each cycle of validation, one part was designated as the unseen test set, and the remaining two were merged and further split into 400 training and 100 validation sets. We trained the model parameters using the training sets and fine-tuned them with the validation sets. The effectiveness of the model was then evaluated using the test sets, which had not been seen by the model during training. A 2D U-Net was employed instead of a 3D architecture due to memory limitations and the relatively large slice thickness of the CT scans (2.5–5 mm), which reduced the benefit of incorporating 3D spatial context. The U-Net-based deep-learning-model used at this stage was developed in Python 3.10.14, using TensorFlow 2.10.0 and Keras 2.10.0. The models were trained on an MSI GeForce RTX 2080 Ti, 11GB RAM, graphical processing unit (GPU). The CT images were provided as training inputs, and the MR labels were assigned as training labels. The U-Net was designed to accept 2D slices of size 512 × 512, from the axial plane of the input brain scan. Once the datasets were organized, we constructed a model with 1 177 649 trainable hyperparameters. We used a batch size of 16, incorporating features such as early stopping and automatic reduction of the learning rate based on the training progress. Data augmentation, including random rotations and translations, was applied during training to enhance model robustness to spatial variability. The model underwent training for 50 epochs. For semantic segmentation, we utilized binary cross-entropy and the Dice coefficient as loss functions to optimize the model's performance. We employed the adaptive moment estimator (Adam) optimizer with a learning rate of 0.00001 to estimate these parameters. All weights were initialized using a normal distribution with a mean of 0 and a standard deviation of 0.01, while all biases were set to zero.

#### Model development using manually derived CT-VCSF labels

In the final stage of the model development, the U-Net-based deep learning models were trained with manual labels derived from CT images. The 62 datasets from the Uppsala University Hospital, consisting of paired CT images and manual lateral CT-VCSF labels, were subdivided into training and cross-validation groups for a three-fold cross-validation (Please refer to the previous section). During each training fold, the 62 paired CT and manual CT-VCSF labels were randomly categorized into training (*n* = 32), testing (*n* = 20), and validation (*n* = 10), groups. To reduce overfitting, we increased the training and validation group sizes using data augmentation.^42^ The U-Net models were developed in Python 3.10.14, using TensorFlow 2.10.0 and Keras 2.10.0, and trained on an MSI GeForce RTX 2080 Ti, 11GB RAM, graphical processing unit. We incorporated the best-performing pre-trained U-Net model, previously trained with automatically derived MR-VCSF labels, by using its encoder weights to initialize the encoders of the current U-Net models. All other parameters, data augmentation, and training conditions remained consistent with our previous models. After adjusting the trainable parameters, the model underwent training propagations within each epoch. These propagation algorithms are iterated for all epochs, with parameters updated accordingly. Training ceased upon model saturation or completion of the specified number of epochs. The resulting models were then preserved for the automated VCSF segmentation of CT scans, leveraging features acquired from both automatically derived MR labels and manually derived CT labels.

### Image processing and analysis

*CT-based volumetric metrics (CTVMs):* Deep-learning-based CT-VCSF segmentation was executed in Python 3.10.14, using TensorFlow 2.10.0 and Keras 2.10.0. The model’s predictions were independent of MRI or any other label or user input, and pre-processing of CT images. The CT images from all cohorts served as inputs to the trained U-Net models. As we used cross-validation, we were able to effectively use the same data (from Gothenburg H70 Birth Cohort studies and Uppsala University Hospital datasets) for both training and testing our models. Cross-validation allowed us to repeatedly train our model on different subsets of the data and then test it on the remaining portions, instead of simply splitting this data into a training set and a separate test set.^42^ Hence, we could use the training inputs as test datasets by applying models to data unseen during the training process. The models efficiently generated lateral VCSF segmentation maps from CT images using the trained hyperparameters, completing the task in approximately 10 s per dataset. Additionally, we derived grey matter, white matter, ICV, and CSF maps using U-Net-based deep learning models trained in our previous study.^34^

The resulting segmentation maps represent probability or confidence maps, with each pixel indicating the likelihood of belonging to the VCSF labels. These maps underwent binarization using global thresholding set at 0.5. Stacking the slices of segmentation maps produced a 3D image, from which the sum of voxels was calculated and then multiplied by the voxel size to determine brain tissue volume (in mL). Total brain volume (BV) was derived from the binarized parenchymal maps. By using the lateral VCSF, CSF, ICV, and BV segmentation maps obtained from CT images via deep learning models, we derived the following iNPH-related CT-based volumetric measures: VCSF/ICV (3D-EI), BV/ICV, and VCSF/CSF.

*Ground truth label volumes:* manually derived CT-VCSF labels and automatically derived MR-VCSF labels were binarized using global thresholding set at 0.5, and volumes were quantified by the sum of voxels, multiplied by the voxel size.

### Statistical analysis

Shapiro–Wilk test was used to examine the Gaussian distribution of the continuous volumetric metrics (*P* > 0.05). The volumetric similarity between automatically derived CT-VCSF and manually derived CT-VCSF in the Uppsala University Hospital datasets, and CT-VCSF and MR-VCSF in the Gothenburg H70 Birth Cohort was assessed using Spearman rank correlation tests, and the agreement was visualized using Bland–Altman plots with 95% limit of agreement. We also computed and compared the Spearman rank correlation between manually and automatically derived CT-VCSF volumes in pre- and post-shunt procedures in the Uppsala University Hospital datasets. We compared the distribution of CTVMs between the iNPH patients from Uppsala University Hospital and controls from Gothenburg H70 Birth Cohort using the Mann–Whitney test (*P* < 0.05). The accuracy of CTVMs in distinguishing iNPH patients from cognitively normal individuals was assessed by measuring the area under the receiver operating characteristic curve (ROC-AUC) with 95% confidence intervals (CI). Additionally, we conducted a comparison of the distribution of CTVMs between iNPH patients and controls, as well as ROC-AUC analysis, on iNPH patients from the validation clinical datasets and Gothenburg H70 Birth Cohort. All statistical analyses and visualization curation were performed using R version 4.3.2 (2023).

### Data and code availability

Data sharing does not apply to this article as no new data were created or analysed in this study. The Gothenburg H70 Birth cohort, Uppsala University Hospital, the Universitätsmedizin Rostock, Germany, and the University of Alabama at Birmingham, United States, cannot openly share data according to existing ethical and data sharing approvals. The analysis pipelines, scripts, and codes used in this study are similarly protected and cannot be shared publicly due to institutional and intellectual property restrictions. Access to relevant data and code can be shared with research groups after submitting a research proposal, which must be approved by the respective study coordinators.

## Results

Table 1 lists the iNPH-related CT-based volumetric measures for all participants in all cohorts. As hypothesized, the mean CT-VCSF/ICV (3D-EI) was higher among the iNPH patients (0.11 ± 0.05) from the validation iNPH patient datasets, as compared to the predominantly healthy Gothenburg H70 Birth Cohort participants (0.04 ± 0.01). Also, the CT-BV/ICV was lower in the iNPH patients (0.65 ± 0.11) in comparison to cognitively normal individuals (0.74 ± 0.03) from the Gothenburg H70 Birth Cohort.

**Table 1:** Summary of iNPH-related CT-based volumetric measures obtained through deep learning

Volumetric metric	Gothenburg H70 birth cohort (*n* = 917)	Uppsala University Hospital iNPH patients (*n* = 62)	Validation clinical iNPH datasets (*n* = 41)	*P*-value (Kruskal–Wallis test)
VCSF/ICV	0.04 ± 0.01	0.11 ± 0.03	0.11 ± 0.05	*P* < 0.001
VCSF/CSF	0.15 ± 0.04	0.35 ± 0.07	0.48 ± 0.13	*P* < 0.001
BV/ICV	0.74 ± 0.03	0.65 ± 0.11	0.71 ± 0.08	*P* < 0.001

Abbreviations: BV, brain volume; CSF, cerebrospinal fluid; CT, computed tomography; ICV, intracranial volume; iNPH, idiopathic normal pressure hydrocephalus; VCSF, lateral ventricular cerebrospinal fluid.

### Segmentation performance compared with manually derived CT-VCSF labels

**Figure 2:**
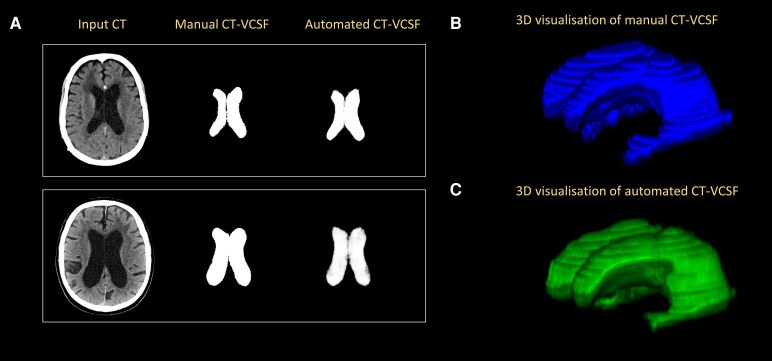
**Model outputs.** (A) Input CT images, manual lateral VCSF, and U-Net predicted lateral VCSF maps of two representative test iNPH patient datasets; (**B**) and (**C**) 3D visualization of manually derived and automatically derived CT-VCSF. Abbreviations: CT, computed tomography; lateral ventricular cerebrospinal fluid, VCSF.

**Figure 3:**
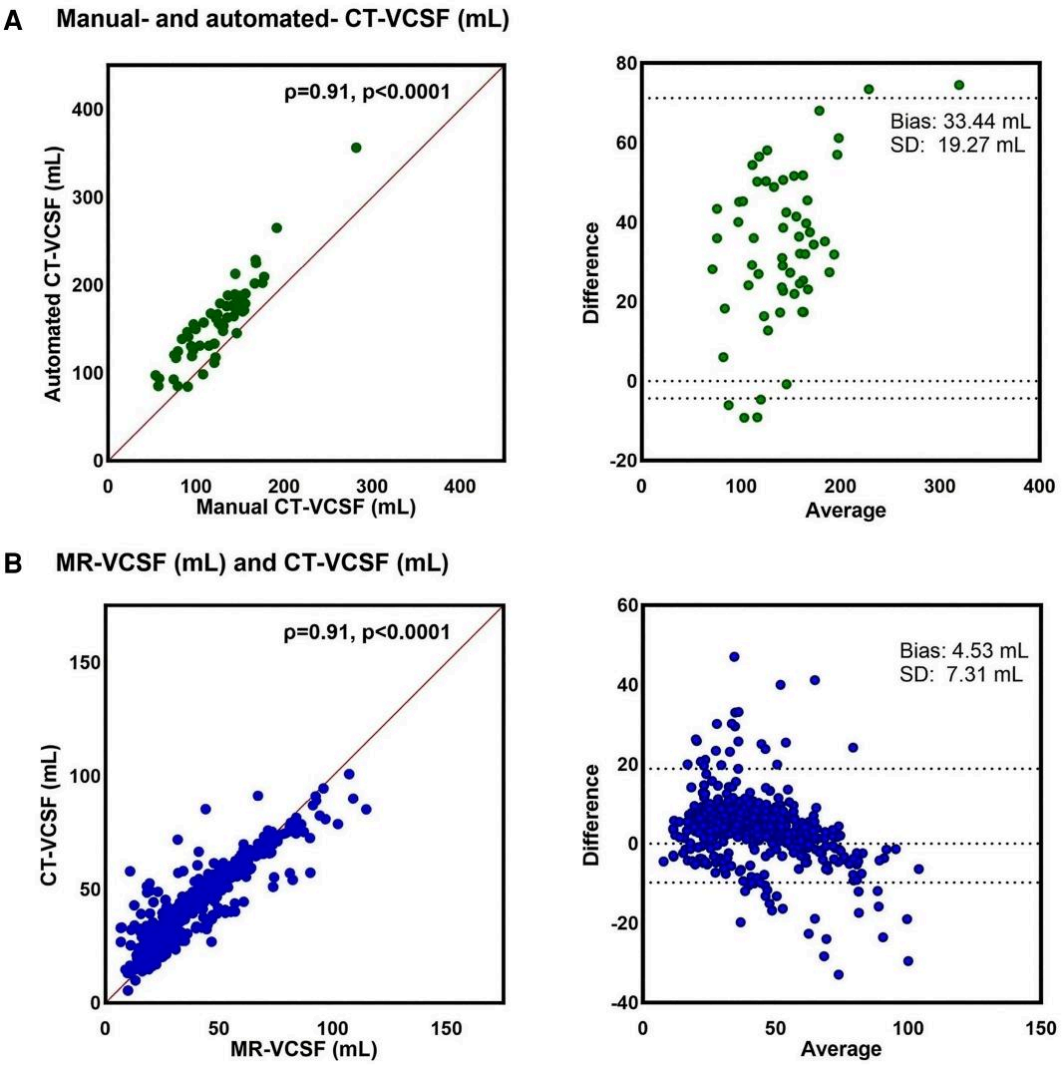
**Segmentation performance for volumetry.** (A) Correlation plots and Bland–Altman analysis plots between manually derived and automatically derived CT-VCSF (*N* = 62); (**B**) Correlation plots and Bland–Altman analysis plots between automatically derived MR- and CT-VCSF (*N* = 734). CT-VCSF predictions for each participant were obtained exclusively from a model that was not trained on that participant’s data, following the three-fold cross-validation rotation scheme described in the Materials and Methods. Each data point represents a single subject’s CT-derived volumetric measure. Abbreviations: CT, computed tomography; MRI: magnetic resonance imaging; SD: standard deviation; lateral ventricular cerebrospinal fluid, VCSF.

Figure 2 illustrates the outputs from our deep learning models, showcasing the predicted CT-VCSF maps alongside their corresponding manual CT-VCSF labels for comparison. In the iNPH patient datasets from the Uppsala University Hospital, automatically derived CT-VCSF volumes demonstrated strong correlations with the manually derived CT-VCSF volumes (*ρ* = 0.91, *P* < 0.001; Fig. 3A, left). The Bland-Altman analysis performed between these two measures yielded a bias of 33.44 ml (24.11*%*) and a standard deviation (SD) of 19.27 ml (Fig. 3A, right) indicating an overestimation through the automatically derived CT-VCSF volumes as compared to manually derived CT-VCSF volumes. Additionally, in the Gothenburg H70 Birth Cohort, CT-VCSF volumes obtained through deep learning demonstrated a strong correlation with the MR-VCSF volumes (*ρ* = 0.91, *P* < 0.001) (Fig. 3B, left), with Bland–Altman analysis indicating a bias of 4.53 ml (14.9*%*) and SD of 7.31 mL (Fig. 3B, right).

### Segmentation performance in the presence of a shunt

**Figure 4:**
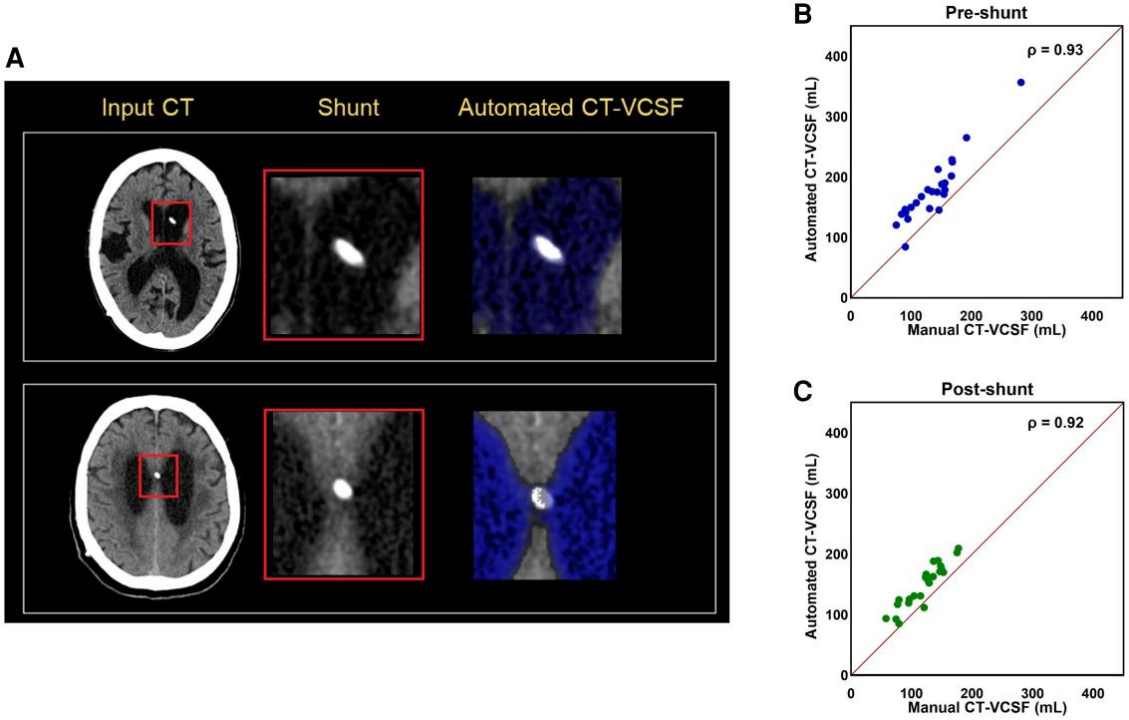
**Model predictions with an intraventricular catheter.** (**A**) Input CT images with shunts, and U-Net predicted VCSF maps of two representative post-shunt iNPH patient test datasets. On the right, correlation plots between manually derived and automatically derived CT-VCSF volumes of (**B**) pre-shunt and (**C**) post-shunt procedure volumes of iNPH patient datasets (N = 23) are depicted. Each data point in (**B**) and (**C**) represents the VCSF volume measurement from a single patient CT scan, comparing manual and automated segmentation results. Abbreviations: CT, computed tomography; lateral ventricular cerebrospinal fluid, VCSF.

The trained deep-learning models effectively predicted segmentation maps, even in the presence of intraventricular catheters. Figure 4A specifically displays the deep learning model’s CT-VCSF predictions at the site of the intraventricular catheter; both pre-and post-shunt VCSF volumetric differences were similar between manual segmentations and those produced automatically by the model, as illustrated in Fig. 4B.

### Diagnostic performance of CTVMs

**Figure 5:**
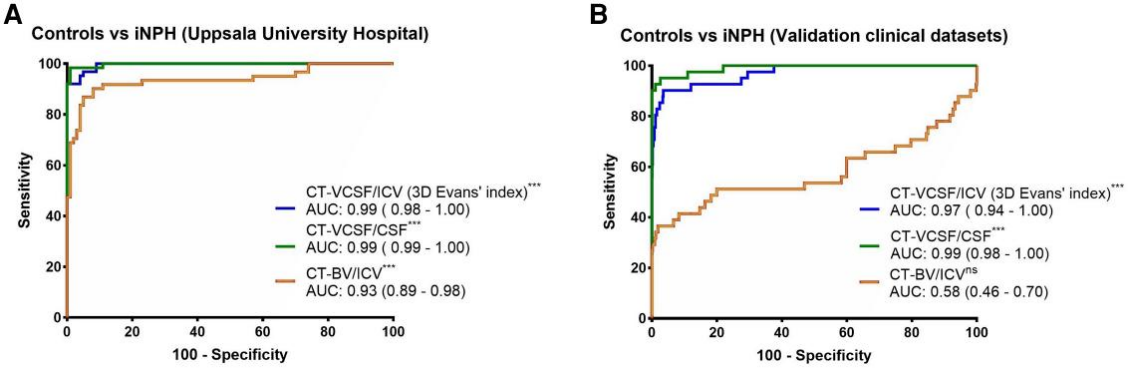
**ROC curves for distinguishing iNPH from controls.** The plot shows the ROC curves of various CTVMs in distinguishing iNPH patients of (**A**) Uppsala University Hospital (*N* = 62) and (**B**) validation clinical datasets (N = 41) from controls obtained from the Gothenburg H70 Birth Cohort (*N* = 917). ***P < 0.001; ^ns^not significant. Abbreviations: brain volume, BV; cerebrospinal fluid, CSF; intracranial volume, ICV; idiopathic normal pressure hydrocephalus, iNPH; lateral ventricular cerebrospinal fluid, VCSF.

**Figure 6:**
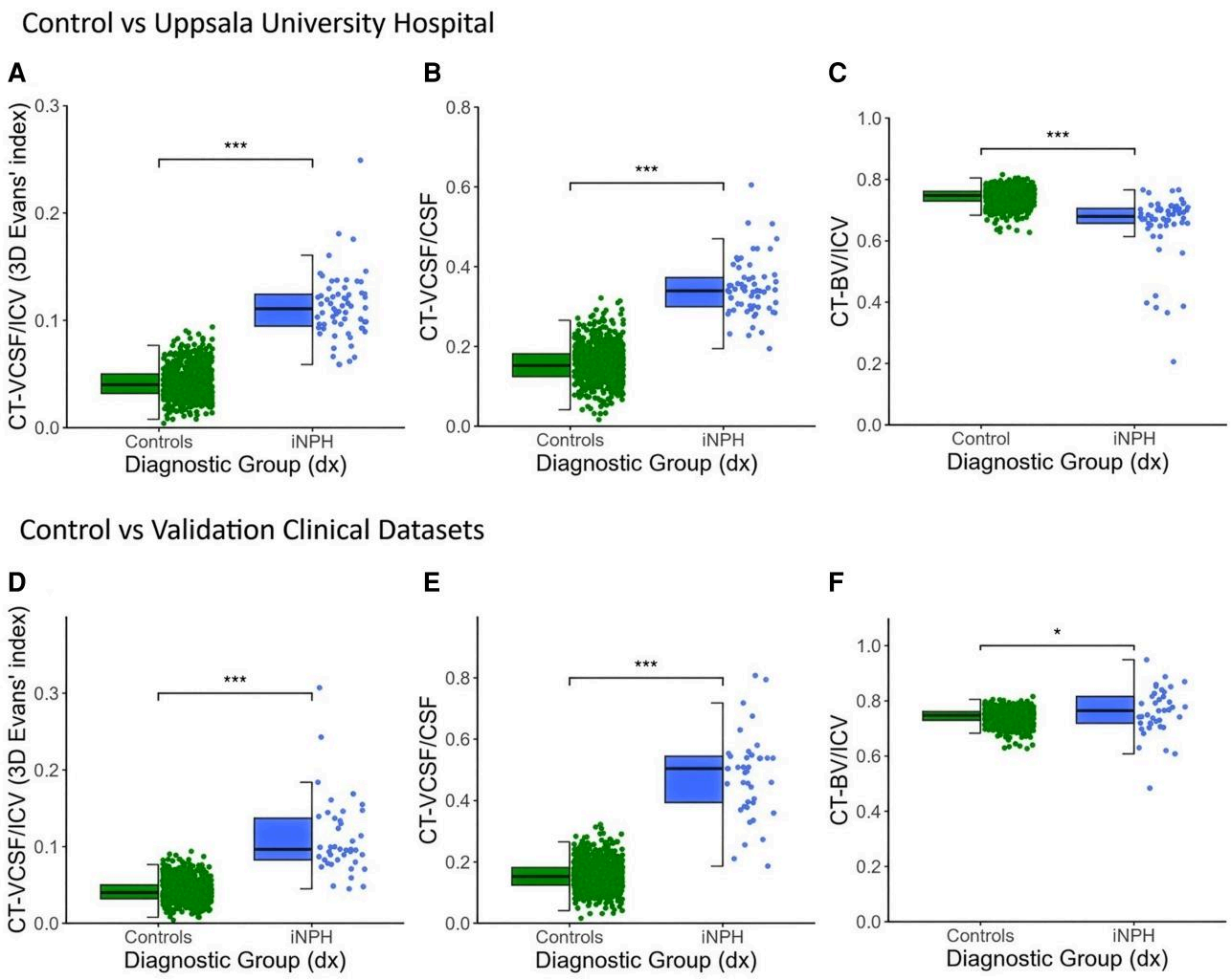
**Distribution of CT-based volumetric measures across iNPH patients.** Panels A–C show iNPH patients from Uppsala University Hospital (*N* = 62), and panels D–F show the corresponding measures in the validation clinical datasets (*N* = 41) and controls from the Gothenburg H70 Birth Cohort (*N* = 917). Each data point represents a single subject’s CT-derived volumetric measure. ***P < 0.001, ^ns^not significant; *P* values are from the Mann–Whitney test. Abbreviations: brain volume, BV; cerebrospinal fluid, CSF; computed tomography, CT; intracranial volume, ICV; idiopathic normal pressure hydrocephalus, iNPH; lateral ventricular cerebrospinal fluid, VCSF.

In the Uppsala University Hospital datasets, iNPH-related CTVMs demonstrated excellent accuracy in distinguishing iNPH patients from controls (datasets from the Gothenburg H70 Birth Cohort), achieving an AUC: 0.99, 95% CI: 0.98, 1.00 for CT-VCSF/ICV (3D-EI) (Fig. 5A). CTVMs significantly differentiated between the two groups, with mean CT-VCSF/ICV (3D-EI) (Fig. 6A), and CT-VCSF/CSF (Fig. 6B) higher in iNPH patients as compared to controls and CT-BV/ICV (Fig. 6C) lower in iNPH patients as compared to controls.

Additionally, in the clinical validation datasets, iNPH-related CTVMs demonstrated high accuracy in distinguishing iNPH patients from controls, with an AUC of 0.97 (95% CI: 0.94 to 1.00) for CT-VCSF/ICV (3D-EI), as shown in Fig. 5B. However, this was not the case for CT-BV/ICV. Overall, CTVMs effectively differentiated between the two groups, with average CT-VCSF/ICV (3D-EI) (Fig. 6D) and CT-VCSF/CSF (Fig. 6E) values being higher in iNPH patients compared to controls and CT-BV/ICV (Fig. 6F) being lower in iNPH patients compared to controls; however, the relationship was not significant.

Supplementary Fig. S1 shows a histogram visualizing the distribution of three CT-based volumetric measures across three different datasets. Each distribution represents one metric measured within a dataset, illustrating variations in volumetric characteristics across samples.

## Discussion

Computed tomography (CT) scans are the major routine method for evaluating brain morphology in both developed and developing countries but typically rely on subjective visual inspection and ratings.^43,44^ Adopting reliable automated and quantitative methods, such as machine learning for ventricular segmentation, could substantially improve the diagnostic performance of CT-derived data through enhanced accuracy and consistency, reducing interrater variability, and providing substantial benefits in both research and clinical contexts. Most machine learning applications for CT scans still rely on time-consuming, labour-intensive, manual labelling, which introduces subjectivity and limits the availability of training datasets.

To address this challenge, our study introduces a pioneering approach using deep learning, trained with both automatically and manually derived brain labels; the standard for evaluating iNPH in research and clinical settings. We utilized automated labels from MRI to enhance training efficiency and address the scarcity of annotated data, which poses significant challenges in brain morphology assessment and quantification. By leveraging transfer learning and automated labels, we aim to improve the accuracy and robustness of our models, mitigating the limitations of manual annotation. Once trained, these models operated solely on CT data, eliminating the need for MRI inputs and/or visual assessments. We successfully applied these models to segment ventricular structures from CT images across two distinct diagnostic groups: a predominantly cognitively normal elderly population (*n* = 917) and iNPH patients (*n* = 94), utilizing data from four different sources, thereby demonstrating the method's robustness and generalizability.

A key finding from our study is that deep learning models, when properly optimized and trained with both automatically and manually derived labels, are highly effective at segmenting VCSF from head CT images with different slice thicknesses (ranging from 3 to 5 mm). Automatically derived CT-VCSF labels strongly correlated with manually derived CT-VCSF (*ρ* = 0.91, *P* < 0.001). However, the bias of 33.44 ml (24.11%) in the Bland–Altman analysis suggests a systematic difference between the two measures, with automatically derived CT-VCSF volumes consistently overestimating lateral ventricular volumes in comparison to manually derived CT-VCSF. The systematic overestimation could potentially enhance the distinction between iNPH patients and controls.

Indeed, the CT-VCSF-based measures derived automatically alone demonstrated a high level of accuracy in differentiating iNPH from control subjects in the current material. Additionally, the automatically derived CT-VCSF measurements showed a strong correlation with automatically derived MR-VCSF, with a bias of 4.53 ml (14.9%) and a standard deviation of 7.37 ml, indicating reasonable agreement albeit with some variability. Congruent with the literature, our segmentation performance was in line with previous work (i.e. that of Zhou *et al*. 2021^32^ and Huff *et al*. 2019^30^). Previously, we confirmed the segmentation similarity between CT brain tissue classes obtained using deep learning and their corresponding MR brain tissue classes determined through established automated segmentation tools.^34^ In this study, we further validated the similarity of CT volume segmentations performed by deep learning against manually derived CT volumes, which currently serve as the gold standard.

Another critical finding of clinical importance from our study was that CT-based volumetric metrics demonstrated high accuracy in distinguishing iNPH patients from cognitively normal individuals in the H70 Cohort, with AUC values of 0.97 and 0.99 for two volumetric ratios: CT-VCSF/ICV (3D-EI) and CT-VCSF/CSF. This was particularly evident in the validation of clinical datasets. In a review by He *et al*. 2020,^45^ the AUC of iNPH versus cognitively normal for manual CT-derived Evans’ index and anteroposterior diameter of the lateral ventricle index were reported to be 0.95 and 0.99, respectively. Compared to MR-derived z-Evans’ index (AUC, 0.91), Evans’ Index (AUC, 0.84), and callosal angle (AUC, 0.93) in Yamada *et al*. 2015,^46^ CT-based volumetric measures exhibited higher diagnostic accuracies. Additionally, in Neikter J *et al*. 2020,^47^ the diagnostic accuracy of MR-derived brain volume ratio (AUC, 0.98), callosal angle (AUC, 0.97), Evans’ Index (AUC, 0.96), and presence of disproportionately enlarged subarachnoid spaces (AUC, 0.96) was comparable to CTVMs. Kockum *et al*. 2020^19^ developed the INPH Radscale, a radiological assessment tool to quantify ventriculomegaly and other brain changes for diagnosing iNPH.^13^ CTVMs also exhibited comparable diagnostic accuracies to iNPH Radscale (AUC, 0.99). A significant advantage of our findings is that the volumetric ratios reported can be automatically extracted within seconds, eliminating the need for time-consuming manual analysis by neuroradiologists. Given the widespread availability of CT scans and their ubiquitous use in clinical routine, the potential for automated screening tools that enhance and expedite radiological assessments is highly promising and is therefore positioned to have an immediate/substantial clinical impact.

Another interesting finding of our study is that the volumetric correlations between automatically and manually derived CT-VCSF volumes in pre-shunt (*ρ* = 0.93, *P* < 0.001) and post-shunt images (*ρ* = 0.92, *P* < 0.001) were similar. This implied that the presence of intraventricular catheters in CT scans had a minimal impact on the model’s segmentation accuracy, as shown in Fig. 4. This consistency underscores the potential for using automated CT ventricular volumes in the ongoing monitoring of iNPH patients after shunt surgery. Such tools could significantly aid in assessing shunt functionality and managing complications related to over- or under-drainage in iNPH and other forms of hydrocephalus.

Given that the model performs well even with the presence of shunt catheters in postoperative scans, it could potentially be used to monitor small volumetric changes associated with shunt dysfunction. Additionally, in elderly patients, enlarged ventricles may represent either hydrocephalus or general brain atrophy,^48^ which diminishes the specificity of traditional metrics like the Evans’ index. Utilizing the specific ratio of brain volume to intracranial volume (BV/ICV) may yield results that are indicative of generalized atrophy, while the ventricular volume to CSF volume (VCSF/CSF) ratio provides a direct assessment of hydrocephalus, highlighting the potential of such an approach as a next-generation tool in clinical settings.

While our findings are promising, several limitations should be acknowledged. Any comparison between a group with established hydrocephalus and healthy controls will result in an exaggerated diagnostic efficiency that is not directly transferable to clinical routine, but rather an early step towards a new diagnostic opportunity. Also, this study focused exclusively on the development and validation of an automated segmentation framework for CT-based volumetric measurement. Although the H70 cohort included cognitively normal individuals, the current analysis did not assess clinical outcomes or cognitive scores, as evaluating clinical associations was outside the scope of this methodological study. Notably, approximately 20% of H70 participants exhibited elevated Evans’ Index values, consistent with known variability in ageing populations; however, their inclusion serves to improve the ecological validity of our model rather than undermine its diagnostic utility. Additionally, the observed variation in BV/ICV across the Uppsala and validation cohorts may reflect underlying anatomical or scanner-related differences and should be explored further in larger, harmonized datasets. Another limitation is the use of Freesurfer-derived segmentations for training, which may introduce variability in BV/ICV estimates, especially in atrophic brains. Our current segmentation framework focuses specifically on ventricular structures and intracranial volume, as subarachnoid space (SAS) segmentation poses greater challenges on routine CT due to the limited soft tissue contrast. While some surrogate features such as VCSF/CSF ratio can partially reflect space distribution, we acknowledge that our pipeline does not explicitly quantify cortical SAS volumes. This could be particularly limiting in cases of disproportionately enlarged subarachnoid space hydrocephalus (DESH), where symptoms may exist despite relatively normal ventricular volumes.

Future research will focus on expanding the clinical utility and robustness of our AI-based CT segmentation tool across the full morphological spectrum of iNPH, as well as in comparison to relevant differential diagnoses such as vascular dementia and parkinsonian syndromes. We also plan to study the relationship between CT-based volumetric measures and MRI-based radiological markers to better understand how automated CT outputs correspond to established diagnostic imaging criteria. Given that ventriculomegaly can occur in asymptomatic individuals, as observed in the H70 cohort, we will further investigate the association between volumetric features and clinical outcomes, including symptom burden and shunt responsiveness. Additionally, while the current framework does not include subarachnoid space segmentation due to CT's limitations in soft tissue contrast, we aim to explore approaches for incorporating SAS metrics using enhanced CT protocols or multimodal (CT-MR) models. These steps are essential for advancing the tool from technical validation towards clinical application in diagnosis, screening, and prognosis.

Taken together, our findings demonstrate that deep-learning-based segmentation of CT images can enable accurate, efficient, and clinically useful volumetric assessments of brain structures in iNPH. These CTVMs not only show strong agreement with manual and MRI-based references but also hold promise as scalable tools to assist the diagnostic work-up and longitudinal monitoring in routine clinical workflows. With further optimization and clinical validation, this approach may serve as a practical and cost-effective alternative to conventional manual analysis and MRI-based methods.

## Supplementary Material

fcag300_Supplementary_Data

## Data Availability

Data can be shared with qualified scientists upon request.
