# Supplementary material for: Assessing CT-based volumetric analysis via deep learning for idiopathic normal pressure hydrocephalus
Source: Brain Commun. 2026 Aug 5;8(4):fcag300. doi: 10.1093/braincomms/fcag300 (PMC13475805; doi:10.1093/braincomms/fcag300)
Supplement: Supplementary file 1 [file fcag300_supplementary_data.docx]

**SUPPLEMENTARY FIGURE**

**Supplementary Figure S1: Histogram of CT-based volumetric measures across all datasets**
